# Differential sperm storage by female zebra finches *Taeniopygia guttata*

**DOI:** 10.1098/rspb.2017.1032

**Published:** 2017-08-16

**Authors:** Nicola Hemmings, Tim Birkhead

**Affiliations:** Department of Animal and Plant Sciences, University of Sheffield, Alfred Denny Building, Western Bank, Sheffield S102TN, UK

**Keywords:** cryptic female choice, post-copulatory sexual selection, sperm competition, sperm storage tubules

## Abstract

When females mate promiscuously, female sperm storage provides scope to bias the fertilization success towards particular males via the non-random acceptance and utilization of sperm. The difficulties observing post-copulatory processes within the female reproductive tract mean that the mechanisms underlying cryptic female choice remain poorly understood. Here, we use zebra finches *Taeniopygia guttata*, selected for divergent sperm lengths, combined with a novel technique for isolating and extracting sperm from avian sperm storage tubules (SSTs), to test the hypothesis that sperm from separate ejaculates are stored differentially by female birds. We show that sperm from different inseminations enter different SSTs in the female reproductive tract, resulting in almost complete segregation of the sperm of competing males. We propose that non-random acceptance of sperm into SSTs, reflected in this case by sperm phenotype, provides a mechanism by which long sperm enjoy enhanced fertilization success in zebra finches.

## 1. Introduction

Darwin's classic view of sexual selection saw males competing for access to females, and females choosing males based on traits expressed prior to copulation [1]. However, our understanding of reproductive behaviour in internal fertilizers has been revolutionized by the realization that females often copulate with more than one male, providing the scope for sexual selection to continue after copulation [2]. In particular, the inevitable delay between insemination and fertilization, often prolonged by a period of female sperm storage in specialized regions of the oviduct, creates the conditions under which sperm from different males can interact and compete [2]. The oviduct may also—via cryptic female choice—provide the means by which females can actively bias fertilization towards certain males through differential sperm acceptance, storage and utilization [3–5]. We currently have limited understanding of the precise mechanisms of cryptic female choice [6], and the extent to which females can exert post-copulatory control over paternity, but it seems very likely that fertilization success is determined by complex interactions between ejaculates and the female tract [7].

In birds, sperm storage is a basic requirement because successive ova are fertilized at intervals of 24 or more hours. Sperm are stored in sperm storage tubules (SSTs) located at the utero-vaginal junction (UVJ) of the oviduct [8], for several days or weeks, depending on species [9]. Avian SSTs are tubular invaginations in the oviduct epithelial tissue. They vary in size within and between individuals and species [10,11], and may or may not be branched [12]. Although undoubtedly specialized for sperm storage, the role of the SSTs in post-copulatory sexual selection remains unknown.

It was once thought that sperm competition success in birds was determined entirely by insemination order, with a last male advantage [13], as occurs in many insects [14]. Later, several mechanisms were proposed to explain this last male advantage in birds [15]: (i) sperm of the last male displaces those of the first, (ii) sperm of different males are stratified, with the last male's sperm overlying those of the first male and therefore leaving the tubule first and (iii) sperm are passively lost from tubules over time, meaning that at any given time there will be fewer sperm from an earlier insemination than a more recent one. Tests of these ideas revealed that passive sperm loss was the main mechanism resulting in a last male effect [16,17]. However, the magnitude of the ‘last male effect’ was exaggerated by the nature of the experimental approach, which involved just two sequential inseminations. In reality, bird copulation patterns are not this simple, and subsequent studies revealed that, in addition to passive sperm loss (that is, the effect of sperm numbers), the outcome of sperm competition is also affected by the ‘quality’ of sperm, including, but not limited to, sperm length, viability and swimming velocity [18–21].

The passive sperm loss model of last male sperm precedence assumes that sperm enter and exit the SSTs at random [15]. However, the mechanisms of storage, maintenance and release of sperm are poorly understood in birds [22,23], and recent evidence suggests that patterns of sperm storage may be non-random and potentially under some degree of female control [24,25].

To fully understand the mechanisms of post-copulatory sexual selection in birds, including the role of the female, we ultimately need to track individual ejaculates within the female reproductive tract. However, labelling ejaculates and/or sperm for identification purposes is a major challenge, particularly in vertebrates. The development of transgenic strains of *Drosphila* that express green fluorescent protein (GFP) and red fluorescent protein in their sperm has revolutionized the study of sperm–female interactions in invertebrates [26,27]. However, the development of stable generations of recombinant vertebrates is more difficult, and the effect of genetic modification on sperm function is unknown. Although germline transgenic GFP birds have been produced [28–31], so far there is no evidence that these birds reliably express GFP in their sperm cells (N. Hemmings 2011, personal observation), or that GFP individuals have equivalent fitness compared with their non-GFP counterparts.

Labelling sperm *in vitro* prior to insemination provides an alternative to genetic modification. King *et al*. [32] used a combination of stained and unstained sperm (from a pooled semen sample from several males) and showed that sperm from two tandem inseminations, 24 h apart, tended to segregate into different SSTs in both the domestic fowl (*Gallus gallus domesticus*) and the turkey (*Meleagris gallopavo*). However, these findings are potentially confounded, because staining may alter the ability of sperm to enter storage. There is little information on the influence of fluorescent stains on sperm viability and motility (see [32], table 1, for a mini-review). King *et al*. [32] found that the nucleic acid dye Hoechst 33342 (Molecular Probes, Eugene, OR) successfully stained domestic fowl and turkey sperm without significantly affecting motility (but see [33,34]) but, surprisingly, Hoechst-stained sperm were *more* likely to be found in SSTs than unstained sperm. As the authors suggest, this unexpected result may have been an artefact: unstained sperm may be difficult to differentiate from stained sperm in the same tubule due to fluorescence glare; the dye may leach and stain unstained sperm and/or the staining process may affect sperm entry or exit from the SSTs. *In vitro* sperm labelling also requires artificial insemination, which itself may alter normal post-copulatory reproductive processes and by-pass mechanisms of cryptic female choice.

If King *et al*.'s [32] results are robust to the confounding issues outlined above, and sperm from different inseminations/males are stored differentially within the SSTs, this would force us to re-evaluate the mechanisms of sperm competition in birds. While the basic effects of passive sperm loss, sperm numbers and sperm quality may be correct, differential sperm storage and utilization would suggest hitherto unrecognized levels of complexity in determining patterns of paternity.

The aim of this study was to test the hypothesis that sperm from different ejaculates are stored differentially within the SSTs of female birds, using lines of zebra finches *Taeniopygia guttata* selected for divergent sperm lengths. The zebra finch exhibits considerable natural inter-male variation in sperm length (mean values for different males vary from approximately 40 to 80 µm [35], but sperm length is extremely consistent both within and between the ejaculates of individual males [36] and highly heritable [37]. Hellriegel & Bernasconi [38] previously used sperm length to investigate differential sperm storage across the three spermathecae in the reproductive tract of female yellow dung flies *Scatophaga stercoraria* (see also [39]), but this has not been previously considered possible in birds due to the fact that avian SSTs are very small, numerous and embedded in female tract epithelial tissue. Here we overcome this issue by using a novel technique to isolate and extract sperm from avian SSTs. This, combined with an experimental mate-switching protocol that allowed natural inseminations by males with different sperm lengths, avoided the potentially confounding effects of artificial insemination and cell labelling on differential sperm storage by females.

## 2. Material and methods

The zebra finches in this study were part of a domesticated population maintained at the University of Sheffield from 1985 to 2016 [35]. Males were from lines that were artificially selected to produce long (greater than 70 µm) or short (less than 60 µm) sperm [24]. The lines have similar sperm numbers and copulation rates, but long sperm have been shown to swim faster and are more likely to fertilize eggs [24]. All males had prior sexual experience, but not with the female they were paired with in this study. Pairs of males whose sperm length differed by more than 12 µm (mean difference + s.e.m. = 19.19 ± 0.52 µm) were chosen to pair with a total of 24 unrelated females (not from the selective breeding lines). The trios of birds and mating orders were selected at the beginning of the study, and during the experiment individuals were identified by unique identification numbers only (which contained no information about their breeding line). This allowed all measurements to be conducted blind with respect to the mating order of long and short males. Females were housed singly in a cage (dimensions 0.6 × 0.5 × 0.4 m) with a nest-box, with an adjoining cage separated by wire mesh to house males during the experiment.

Following the experimental mate-switching protocol of Bennison *et al*. [24], one male was paired to the female for 3 days (and allowed to copulate freely), then replaced with the second male for a further 3 days. The second male was then placed in the adjoining cage with a wire mesh division to prevent further physical contact. The pairing order of long- and short-sperm males was alternated across females. Copulation rate was not recorded in this study, but previous work has shown the long- and short-sperm males attempt copulations at a similar rate, and females do not appear to preferentially accept copulations from either long- or short-sperm males [24].

The first egg laid by each female was removed from the nest on the morning of laying and examined for the presence of sperm as described in Birkhead *et al*. [40]. The perivitelline layer was examined for sperm under 400× magnification using darkfield microscopy (Leica DMBL). Sperm were imaged using an Infinity 3 camera (Luminera Corporation) and Infinity Analyse software, and sperm length was measured to the nearest 0.01 µm to determine whether each sperm was from the long- or short-sperm producing male.

If sperm from both males were present on the perivitelline layer of the first egg laid, the female was humanely killed by cervical dislocation on the same day (under Schedule 1 (Animals (Scientific Procedures) Act 1986)), and immediately dissected to remove the oviduct. Of the 24 females used in this experiment, 13 produced eggs on whose pervitelline layers the sperm from both males was present. Mating order was not evenly split across these females: five were paired with the short-sperm producing male first and eight were paired with the long-sperm producing male first. Of the remaining 11 females, two did not produce any eggs, two produced unfertilized eggs with no sperm present on the perivitelline layer and seven produced eggs that had been reached by sperm from one male only. Since in the latter cases we could not be sure that both males had inseminated these females, they were not dissected to avoid unnecessary sacrifice of birds. The second ovum, which in all cases had been recently ovulated and was located in the magnum or isthmus of the oviduct, was removed so that the PVL could be examined (as above) and the proportion of PVL sperm from each male compared with that stored in the SSTs.

To isolate the SSTs, the oviduct was first cut longitudinally and opened out flat, and four non-adjacent, equally spaced primary mucosal folds were removed. Folds were kept on ice in phosphate-buffered saline until dissection. Each mucosal fold was spread out on a glass slide with 10 µl PBS and examined under an SMZ25 stereomicroscope with 2× objective lens and 15.75× zoom to locate the UVJ and SSTs. Using fine, stainless steel insect pins, the mucosal tissue was gently separated into small segments surrounding any individual SSTs that contained sperm, taking care not to distort or damage the structure of the SST itself. Once a segment was isolated, it was transferred using the tip of a pin to a 5 µl drop of clean PBS, and the SST was then pulled open to release the sperm inside (electronic supplementary material, figure S1). The longitudinal region of the UVJ (i.e. vagina end, middle or uterus end) from which each SST was isolated was recorded. If the UVJ was particularly small, it was divided into vagina and uterus end only. A coverslip was immediately placed on the top of the sample and the slide was then examined at 400× magnification using darkfield microscopy (Leica DMBL). All sperm from each sample were imaged using an Infinity 3 camera (Luminera Corporation) and Infinity Analyse software, and sperm length was measured to the nearest 0.01 µm to determine whether each sperm was from the long- or short-sperm producing male. An overall count of long and short sperm was obtained for each SST. In a very small number of cases (33 (0.6%) of 5398 sperm examined), the sperm cell was damaged in the process of dissection and, therefore, it was not possible to ascertain whether it was long or short. These sperm were excluded from our analyses.

As many sperm-containing SSTs as possible from each mucosal fold were analysed within a 30-min period. The total time between the initial dissection of the mucosal folds and isolation of the final SST was limited to 2 h, because beyond this time the tissue began to degrade. In total, 644 SSTs from the 13 females were examined; these were not evenly spread across all females due to differences in sperm numbers stored, with 8–104 SSTs examined per female. Data for each female are summarized in the electronic supplementary material, table S1.

Almost all SSTs were occupied by either short or long sperm only, and the proportion of SSTs containing either short or long sperm was highly correlated with the total proportion of short or long sperm stored across all SSTs (see Results). We, therefore, created a binary response variable by classifying each SST as either ‘long sperm’ or ‘short sperm’ (excluding a single SST in which both short and long sperm were found; see Results). The likelihood of an SST being occupied by short or long sperm was then analysed as a function of mating order (i.e. whether the long-sperm or short-sperm producing male was paired with the female first), the region of the UVJ in which the SST was located, and the interaction between these two variables, using a generalized linear mixed model, with mating trio included as a random effect (all individuals were included in one mating trio only) and a binomial error distribution. The analysis was carried out using the *glmer* function (library *lme4*) in R v. 3.3.1 [41].

## 3. Results

Only one of 664 SSTs examined, across 13 females, contained sperm from both males. Therefore, in 99.8% of SSTs, and 12 of 13 females, sperm from different males were completely segregated in storage. The single tubule where sperm from both males were found was branched (across female zebra finches, 4–27% of tubules are branched [11], and this was typical in the present study), and sperm from the long-sperm producing male were located in a separate branch of the tubule to sperm from the short-sperm producing male, therefore maintaining a degree of inter-male segregation.

The absolute proportion of each male's sperm stored by the female (based on a total sperm count across all tubules examined) was highly positively correlated with the proportion of SSTs containing each male's sperm (Pearson's product-moment correlation = 0.963, *t* = 11.867, d.f. =11, *p* < 0.001). This indicates that both long and short sperm were aggregated to a similar degree across SSTs, and confirms that the number of SSTs occupied by each sperm morph is a good proxy for the overall number of each sperm morph stored. In addition, the proportion of each male's sperm found on the PVL of the female's second ovum (taken from the oviduct during dissection) was significantly positively correlated with the proportion in storage (Pearson's product-moment correlation = 0.857, *t* = 5.526, d.f. = 11, *p* < 0.001).

**Figure 1.**
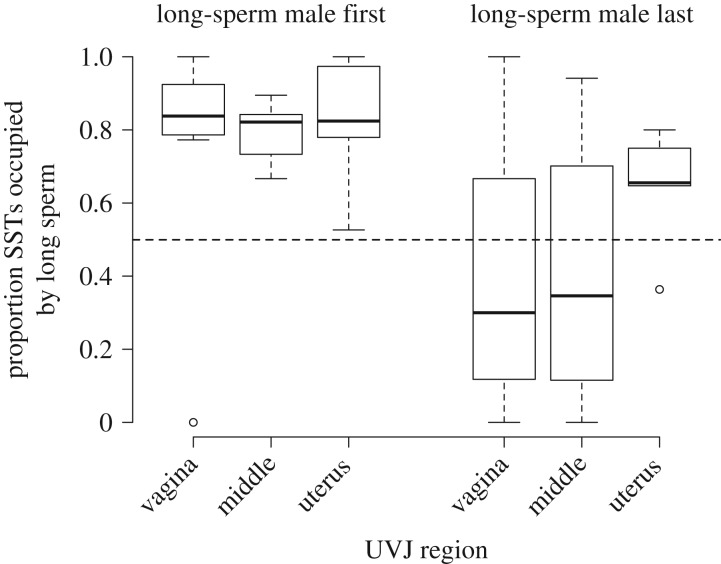
The proportion of SSTs occupied by long sperm across different regions of the UVJ, when female zebra finches copulate sequentially with both a short-sperm and long-sperm producing male (data separated by mating order). Data are from 643 SSTs, all of which were occupied by long sperm or short sperm only (no mixing), across 13 females.

Overall, SSTs containing sperm from the long-sperm producing male were more common across the entire UVJ, with 68.43 ± 7.39% (mean ± s.e.m.) of SSTs containing long sperm across the 13 females examined (*X*^2^ = 127.32, d.f. = 12, *p* < 0.001). However, the long-sperm advantage was particularly notable when long-sperm producing males were the first to copulate (estimated effect = 2.177 ± 0.726, *z* = 3.000, *p* = 0.003; data from 643 SSTs across 13 females; figure 1).

There was also evidence that SSTs containing long or short sperm were spatially segregated across the regions of the UVJ, but this effect depended on mating order (i.e. whether the long-sperm producing male copulated first or last). Sperm from long-sperm producing males were significantly more likely to occupy SSTs in the uterus region of the UVJ (but not the vagina or middle regions) when the long-sperm male was last to copulate, compared to when he was first (estimated effect of the interaction between mating order and UVJ region = 1.366, *z* = 2.612, *p* = 0.009; analysis based on data from 643 SSTs across 13 females; figure 1).

## 4. Discussion

We have shown that, following sequential inseminations, sperm from different males, with different sperm lengths, do not mix in in the SSTs of female zebra finches, but instead enter different tubules. Virtually no tubules examined (0.2%) contained sperm from both males, supporting our hypothesis that sperm from different ejaculates are stored differentially across the SSTs of female birds. Indeed, this is probably a conservative estimate of the degree of segregation, because we did not examine any females whose eggs had been reached by sperm from only one male (these females were probably inseminated by both males, but exhibited more extreme differential sperm storage than the females included in our analyses). Importantly, our results demonstrate that sperm entry into SSTs is non-random, refuting a key assumption of the passive sperm loss model of sperm competition in birds. Our data also show that the proportions of sperm from each male in storage are representative of those reaching the site of fertilization (i.e. trapped on the ovum PVL), providing convincing support for the assumption that most sperm selection occurs in the vagina, before entering the SSTs, and that the tiny (approx. 1%) proportion of inseminated sperm reaching the ovum comprise a random sample of those from the SSTs [22].

Identifying sperm from different males by length allowed us to examine the evidence for differential female sperm storage following natural copulations, without the potentially confounding effects of sperm labelling and artificial insemination that has limited other studies (e.g. [32]). The fact that long sperm were more likely to be stored by female zebra finches is consistent with our earlier findings that long sperm have greater success in reaching and fertilizing ova in this species, which we assume to be due to their superior swimming ability [24]. However, our present study reveals that the strength of this long-sperm advantage depends on male mating order: if long-sperm males were *first* to inseminate the female, their sperm were more likely to occupy SSTs, but this advantage was reduced when they were last to copulate (figure 1). An exception to this pattern was seen for SSTs located in the uterus region of the UVJ. In this region, furthest from the site of insemination, long sperm were more likely to be stored regardless of male mating order.

While our experimental protocol avoided the potential confounding effects of artificial insemination and sperm labelling, there are some limitations to what we can infer from our results. We do not know the extent to which sperm segregation is caused by sperm length *per se*, as opposed to the fact that sperm belong to two different males. In addition, we cannot conclusively say that sperm from different males—as opposed to different ejaculates—are segregated. If a female copulates with a single male only, it is possible that sperm from his successive inseminations might also be stored in different SSTs, depending on the mechanism(s) underlying sperm segregation. One possibility is that SSTs become unreceptive or ‘closed’ some time after an initial set of sperm enters, and if this is the case, then entry of subsequent sperm will be prevented regardless of which male they are from. Alternatively, sperm and/or seminal fluid from one male may have a repellent effect on sperm from another male (e.g. via chemical cues); in this case, sperm from successive inseminations by the same male would not be expected to segregate. To explicitly test whether segregation is due to sperm being from different males, one would have to simultaneously inseminate sperm from two different males with similar sperm lengths, thereby removing the confounding effects of sperm length and insemination interval. However, current methodologies would not allow us to distinguish sperm from these two males. Deducing the precise mechanisms underlying sperm segregation is therefore an important avenue for future study.

If sperm segregation is driven by a temporal change in SST receptivity, we might expect the ‘block’ to occur relatively slowly, because the mechanisms underlying sperm acceptance and release from SSTs are at least partially under hormonal control [23,25]. In preliminary trials, where our mate-switching interval was reduced from 3 days to either (i) 1 h or (ii) 1 day (electronic supplementary material, S2), sperm from different males were predominantly segregated (as in the 3 day interval trials reported here), but the occurrence of sperm mixing within tubules was slightly more frequent (electronic supplementary material, table S2). However, owing to a high rate of second male rejection in these preliminary trials, only three females produced eggs with sperm from both males (one female after the 1-h mating interval, and two after the 24-h mating interval), so we did not have sufficient data to further explore the possibility that mating interval influences the likelihood of differential sperm storage.

If sperm are unable to enter already-occupied SSTs (after a certain time interval), this may provide an explanation for the apparent precedence of first-male sperm that we observed in most regions of the UVJ. Once the sperm of the first male to copulate are residing inside a tubule, it may not be possible for sperm from subsequent males to enter. However, the underlying first-male advantage that we (and [24]) found could also simply result from a female preference for the first male she is paired with. The fact that copulations from second males were often rejected (see above and electronic supplementary material, S2) is consistent with this idea.

In both this study and that of Bennison *et al*. [24], the first-male advantage outlined above was less dramatic for short-sperm producing males (figure 1). This may be due to the relatively poor swimming ability of short sperm: in order to reach the SSTs, sperm must traverse the hostile environment of the vagina, and short sperm swim more slowly than long sperm in this species [24]. It is therefore plausible that fewer short sperm are able to reach the SSTs regardless of the competitive scenario. If this were the case, we would also expect the disparity in success of short sperm and long sperm to become more marked as we move along the UVJ, which is exactly what we found. When the long-sperm producing male was last to mate, SSTs in the uterus region of the UVJ (i.e. furthest from the site of insemination) were more likely to contain long sperm than short sperm, compared to those in the vagina or middle regions (figure 1). If SST receptivity does change in response to sperm occupancy, as hypothesized above, it is possible that when short-sperm males copulate first, their sperm reach and enter SSTs located at the most proximate regions of the UVJ (vagina and middle), rendering these inaccessible to sperm from long-sperm males that copulate afterwards. However, as suggested above, the slower swimming short sperm may be less able to access the uterus end of the UVJ, leaving more SSTs in that region free for the second male's longer and faster sperm to populate.

In summary, we have demonstrated that sperm from different males are differentially stored in the SSTs of female zebra finches. We know that the outcome of sperm competition in birds is determined by a combination of passive sperm loss, sperm numbers and sperm phenotype (in this case, sperm length, which is closely correlated with swimming velocity). Non-random acceptance of sperm into SSTs, mediated by sperm phenotype, may provide the mechanism by which long sperm enjoy enhanced fertilization success in zebra finches.

## Supplementary Material

Supplementary Material 1; Supplementary Material 2

## Data accessibility

Data are available via Dryad (http://dx.doi.org/10.5061/dryad.46bq0) [42].
