# Supplementary material for: Differential sperm storage by female zebra finches Taeniopygia guttata
Source: Proc Biol Sci. 2017 Aug 16;284(1860):20171032. doi: 10.1098/rspb.2017.1032 (PMC5563812; doi:10.1098/rspb.2017.1032)
Supplement: Supplementary Material 1; Supplementary Material 2 [file rspb20171032supp1.pdf]

Differential sperm storage by female zebra finches *Taeniopygia guttata*

Supplementary Material

Nicola Hemmings, Tim Birkhead

Supplementary Material 1: Additional sperm storage tubule information

Figure S1. (A) Zebra finch uterovaginal junction epithelial tissue showing sperm storage tubules with and without sperm; photo taken under a Nikon SMZ25 stereomicroscope with 2x objective lens and 15.75x zoom. (B) Sperm storage tubules isolated from epithelial tissue; photo taken under a Nikon SMZ25 stereomicroscope with 2x objective lens and 15.75x zoom. (C) Zebra finch sperm released from a tubule; photo taken under 400x darkfield microscopy (Leica DMBL).

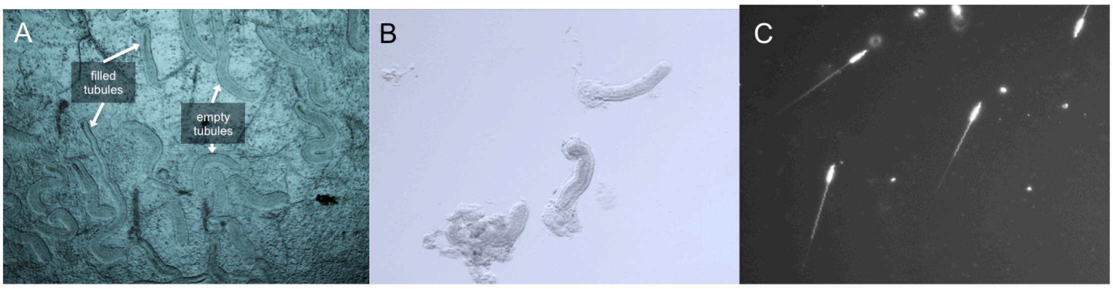

Table S1. Numbers of sperm storage tubules (SSTs) examined per female

mating order	vaginal region	mid-region[1]	uterine region	total
short male first	3	-	5	8
(n = 5 females)	9	25	11	45
	20	13	12	45
	21	17	17	55
	17	26	29	72
long male first	5	-	5	10
(n = 7 females)	5	-	7	12
	11	-	10	21
	15	19	19	53
	22	21	19	62
	22	19	32	73
	33	28	22	83
	35	30	38	103

[1] If the utero-vaginal junction was particularly short it was divided into vagina and uterus regions only.

Supplementary Material 2: Reduced copulation interval trials – methods and results.

Methods

Using an additional 15 females, we attempted to reduce the interval between copulations by the long and short-sperm producing males to test whether sperm segregation was related to the timing of inseminations.

One-hour copulation interval

First, we attempted to reduce the copulation interval to one hour as follows. Females were set up with one male in a double cage, with a wire mesh divider extending through the nest box, to prevent copulation. Having the male present behind the wire divider was necessary to induce egg laying by the female. On the day the first egg was laid, the male was placed into the female's side of the cage and the pair was observed until copulation took place. If a copulation was observed, the male was removed from the experimental room, and after one hour, replaced with another male from the opposite sperm length line. The pair was again observed to confirm copulation occurred (for up to a maximum of one hour), after which the second male was removed and the original male returned behind the wire divider to encourage the female to continue laying. If both males were observed to copulate with the female, she was dissected the following day to examine the sperm in her SSTs following the methods described in the main manuscript text. Unfortunately, in only one case was the female observed to copulate with both males under this experimental set up, mainly due to a lack of acceptance of the second male by the female.

24-hour copulation interval

Since the one-hour copulation interval trials were unsuccessful, we next attempted to orchestrate 24-hour copulation interval trials with the remaining 14 females as follows. After a period of 14 days (the maximum sperm storage duration in the zebra finch), the females were once again set up with one male in a double cage, with a wire mesh division that extended between the nest box, to prevent them from copulating. Pairs were left like this for 4 days, to allow females to initiate another breeding cycle. On day 5, the male was placed into the female's side of the cage and the pair was left to copulate freely for 24 hours. After 24 hours, the first male was removed and replaced with a second male (from the opposite sperm-length line) for an additional 24 hours. The second male was then placed behind the wire divider to prevent further copulations. Of the 14 females included in this trial, only 2 produced eggs with sperm from both males present on the PVL. These two females were dissected on the first day of egg laying to examine their SSTs following the methods described in the main text.

Results

One-hour copulation interval

A total of 115 SSTs was examined across 4 primary mucosal folds from the single female examined in this trial. Of these, 18 contained short sperm only, 77 contained long sperm only, and 19 contained both short and long sperm (see Table S2). One other SST contained a large clump of sperm that could not be dispersed and therefore it was not possible to distinguish between long and short sperm with confidence. Excluding this single SST, 17% of SSTs contained sperm from both males in this female, indicating a higher degree of sperm "mixing" than found in the 3-day copulation interval trials. Of the 19 SSTs containing both males' sperm, long and short sperm were segregated either into different branches or different ends of the tubule in 6 (32%); in the other 13 SSTs, long and short sperm occupied the same region of the tubule.

24-hour copulation interval

Summary data from the two females examined in this trial are provided in Table S2. In the first female, sperm from both males were found in 7 of 42 (17%) SSTs examined, and in 4 of these 7 SSTs, sperm from different males were segregated into different branches or ends of the tubule. Again, this indicates a higher degree of sperm "mixing" than found in the 3-day copulation interval trials. However, in the second female, sperm from the two males were completely segregated across tubules (n = 35), with no sperm mixing observed whatsoever. Therefore, across both females from the 24-hour copulation interval trial, sperm from different males were completely segregated in 91% of tubules examined.

Table S2. Summary data from reduced copulation interval trials

copulation interval	no. SSTs examined	% long sperm SSTs	% short sperm SSTs	% mixed sperm SSTs
1 hour (n = 1 female, long male first)	114[1]	68	16	17
24 hours (n = 2 females, short male first)	42<br>35	69<br>57	14<br>43	17<br>0

[1]A total of 115 SSTs was examined but one SST is excluded because sperm couldn't be measured with confidence due to clumping.
